# Epidemiology of measles in Oromia region, Ethiopia, 2007-2016

**DOI:** 10.11604/pamj.2020.37.171.23543

**Published:** 2020-10-20

**Authors:** Mulugeta Asefa Gutu, Alemayehu Bekele, Yimer Seid, Abyot Bekele Woyessa

**Affiliations:** 1 Ethiopian Public Health Institute, Addis Ababa, Ethiopia,; 2 Ethiopian Public Health Association, Addis Ababa, Ethiopia,; 3 Addis Ababa University School of Public Health, Addis Ababa, Ethiopia

**Keywords:** Data, measles, surveillance, Oromia

## Abstract

**Introduction:**

measles is the leading vaccine preventable childhood disease designated for elimination by WHO. More than 20 million people are affected by measles each year, particularly in Africa and Asia. With annual outbreaks reported from Ethiopia´s Oromia region. We analyzed measles containing vaccine coverage (MCV), measles cases and measles deaths over a 10-year period (2007-2016).

**Methods:**

we reviewed Oromia measles surveillance data and first-dose measles containing vaccine (MCV1) administrative coverage. Descriptive statistics and multivariable logistic regression were performed to assess variables associated with measles death. Additional spatial mapping was performed to visually display key areas of measles case distribution in Oromia.

**Results:**

a total of 26,908 measles suspect cases were identified, of which 18,223 (68%) were confirmed. A median age of 6 years (IQ range 0.5-71 years) and 288 deaths were observed. Among the total cases, 29% were unvaccinated and 46% had unknown vaccination status. The highest IR was seen in Guji zone (IR=190/100,000 population) among 1-4 years, with a majority from rural areas. Risk factors associated with death include age <5 years (AOR=1.82, CI: 1.42-2.33), unvaccinated status (AOR=1.44, CI: 1.06-1.95) and inpatient treatment (AOR=2.12, CI: 1.58-2.85). Of 8,732 measles IgM negative/indeterminate specimens, 10.5% tested positive for rubella specific IgM.

**Conclusion:**

outbreaks of measles are an ongoing public health concern in the Oromia region. Children aged 1-15 years remain at high risk for contracting measles in the region. We recommend strengthening routine immunization to reach all children, especially in rural areas and that the measles-rubella (MR) vaccine be considered.

## Introduction

Measles, caused by measles virus, is a highly infectious viral disease. Humans serve as the only natural hosts of the virus [1], with symptoms typically manifesting within 10-12 days after exposure to an infected person and typically lasting for 7-10 days. Transmission occurs primarily by airborne respiratory droplets to mucous membranes in the upper respiratory tract or the conjunctiva. It is estimated that approximately 30 percent of reported measles cases may have one or more complications and these occur most commonly among children younger than 5 years of age and adults 20 years of age and older [2]. Measles occurs worldwide and despite the existence of effective vaccine, remains a significant cause of childhood morbidity and mortality. The disease kills more children than any other vaccine-preventable disease [3]. According to the Centers for Disease Control and Prevention (CDC), in 2004, it was estimated that global measles mortality was 454,000, of which 410,000 were children under the age of 5 [4]. Similarly, the World Health Organization (WHO) reported approximately 110,000 measles deaths globally in 2017. Once again, the majority of these deaths involved children under the age of 5 years, regardless of the availability of a safe and effective vaccine [5].

In 2000, the measles burden was composed of an estimated 39.9 million measles cases, 777,000 deaths and 28 million disability-adjusted life years. This pathogen has particularly impacted the World Health Organization regions of Africa and South-East Asia, which were responsible for 70% of these incident cases and 84% of measles-related deaths [6]. To combat this burden, starting in 2001, countries in the WHO Africa region began implementing the recommended measles control strategies and by 2011 had adopted the regional elimination goal [7]. Guiding this process were three milestones towards elimination set by WHO to be met by 2015: increase routine coverage with the 1^st^ dose of a measles-containing vaccine (MCV1) among children aged 1 year to ≥90% nationally and exceed 80% vaccination coverage in every district or equivalent administrative unit; reduce annual measles incidence to less than five cases per million and maintain that level; reduce measles mortality by 95% or more in comparison with 2000 estimates [8]. Although significant improvements were seen these goals were not met [9] and further goals were set in place to be achieved by the end of 2020 [10]. The most recent estimates from 2017 still depict a serious issue with 6.7 million cases and 110,000 deaths [6]. In many developing countries measles remains the most important cause of death between the ages of one and five years [11] and has demonstrated a shift in the age specific incidence to a higher age [10].

In Ethiopia, measles has been one of the major causes of death and sickness of children and it is among the notifiable lists of diseases in the country. The 1^st^ dose of measles containing vaccine (MCV1) is given at the age of 9 months, with the second opportunity only available through supplementary immunization activities [12]. In 2019 the country introduced a second dose into the routine schedule [13]. Between 1^st^ January and 31^st^ March 2017, 348 cases were confirmed and 40 outbreaks reported in Ethiopia´s Addis Ababa, Afar, Amhara, Oromia, Southern Nations Nationalities and Peoples, Somali and Tigray regions. Of particular concern in Ethiopia is the Oromia region where outbreaks of measles are reported each year [14]. Oromia is one of 9 regional states in Ethiopia. Which has 18 administrative zones and 304 districts. According to Central Statics Agency (CSA) population and housing 2007 census, projected for 2016, Oromia region has an estimated 35,127,213 people [15]. Over a 5 year period, from 2005-2009, 3,507 suspected cases of measles were reported from this region alone [16]. We aimed to assess the epidemiology of measles within the region and to describe its distribution in Oromia from 2007-2016, review MCV1 vaccination coverage and to assess variables associated with death.

## Methods

We conducted cross-sectional retrospective descriptive data analysis of measles surveillance reported data from 2007-2016 in Oromia region. We extracted 10 years (2007-2016) of Oromia measles surveillance data from the Ethiopian Public Health Institute (EPHI) national surveillance database. Cases were collected from standard case-based investigation forms or through a line list table that summarizes information about persons who may be associated with an outbreak. We defined a suspected measles case as any person with fever and maculopapular (non-vesicular) generalized rash and cough, coryza or conjunctivitis (red eyes). We further classified the suspected measles cases into four categories, laboratory confirmed, epidemiologically linked, clinical/compatible and discarded, as per the national and WHO guidelines [17]. The sum of laboratory confirmed, clinically compatible and epidemiologically confirmed cases were considered as measles.

**Data processing and analysis:** the obtained data was cleaned and checked for duplication by patient name, sex, age, residence area, date of onset, seen at health facility and date sample collection. Statistical analysis was performed using Microsoft Excel. Descriptive statistics including frequency, percentage, rate and ratio were calculated. We further calculated cumulative age, sex and area specific measles incidence rates using 2007 population census as denominator. We divided the number of measles cases to population and multiplied by 100,000 to calculate cumulative reporting rate. Age, year and area specific measles cases were divided to respective population and multiplied by 100,000 to calculate specific IR. We also divided the average of ten years measles cases to mid-year population and multiplied by 100,000 to calculate annualized incidence rate. Non-measles febrile rash case rate was determined by dividing laboratory negative suspected measles cases to population and multiplying by 100,000. Bivariate and multivariate logistic regression was calculated to assess variables associated with measles death. Crude and adjusted odds ratios (AOR) and their 95% confidence intervals (CI) were calculated. We used ArcGIS 10.4.1 software to illustrate the study area with measles case distribution.

## Results

**Figure 1:**
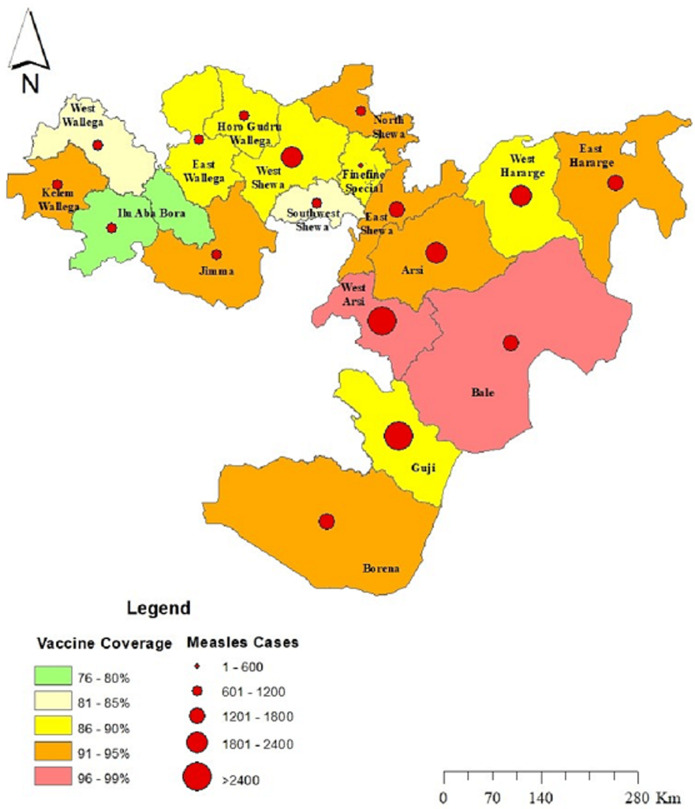
map Oromia region by category of measles distribution from 2007-2016 and five year average of routine measles vaccination coverage from 2012-2017

**Figure 2:**
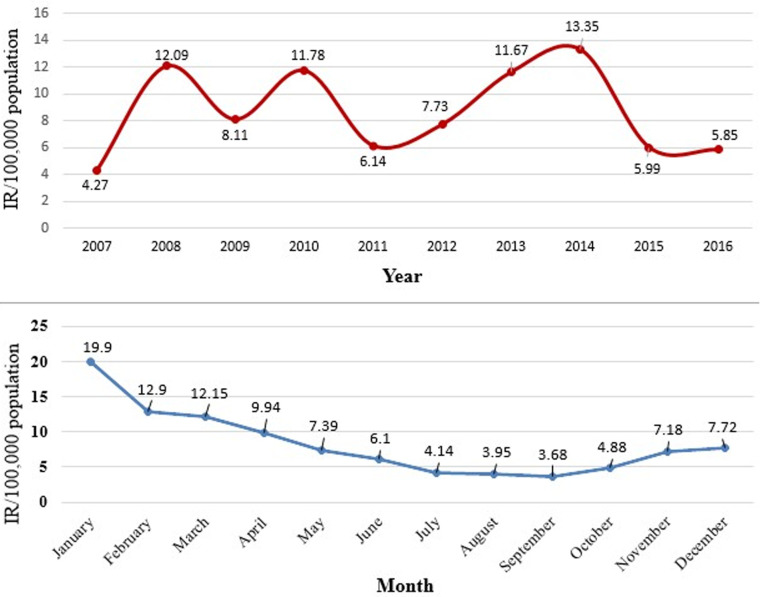
trend of measles incidence rates by year/month, Oromia region, 2007-2016

Measles was seen in all Woredas in Oromia (Figure 1). A total of 26,908 suspected measles cases and 288 deaths were reported in Oromia region from 1^st^ January 2007 to 31^st^ December 2016. Measles annual incidence rate (IR) was 6.02 per 100,000 population with an overall case fatality rate (CFR) of 10.7 per 1000 population. The median age was 6.0 years and IQR was from 6 month to 71 years. Males were most affected (IR=9/100,000 population), with a female to male ratio of 1: 1.1. Overall 17,236/26,908 (64%) of suspected measles cases were reported by case-based reporting, while 9,672/26,908 (36%) were reported by line list. The majority (55%) of case-based reports occurred during 2012-2016, as compared to (45%) during 2007-2011. There was no report by line list during years 2011 and 2015. The majority of cases, 21,240/26,908 (79%), occurred in rural areas. The majority of cases were treated outpatient level, 23,328/26,908 (88%), and the admission rate was (12%). Of total reported suspected measles cases, age-specific IR showed 1-4 year most affected (IR=27/100,000 population), followed by <1 year (IR=26/100,000 population) and 5-14 years (IR=12 /100,000 population), while lowest IR was >44 year age group (IR=0.003 /100,000). Similar trends were seen for CFR, with the highest death among 1-4 year CFR (1.4%), followed by <1 year CFR (1.3%), while lowest CFR were >44 years CFR (0.000%). Higher CFR 222/288 (77%) were observed during 2012-2016 as compared to 66/288 (23%) during 2007-2011 years. The highest IRs were observed in 2014 (IR=13/100,000) followed by 2008 (IR=12/100,000 populations), while the lowest IR were seen in 2007 (IR=4 /100,000 populations) (Figure 2). Highest CFR was also reported in 2014 (CFR=30/1,000 population), followed by 2013 (CFR=20 per 1,000 population). Cases were highest during the first quarter (January to March) 12,116/26,908 (45%) followed by second quarter (April-June) 6,305/26,908 (23.4%), third quarter (August-September) 3,167/26,908 (12.7%) and fourth quarter (October-December) 5320/26908 (19.8%) (Figure 2). Regionally, Guji zone was most affected (IR=190/100,000 population) followed by West Arsi zone (IR=170/100,000 population), while Adama town (IR=20/100,000 population) was least affected.

Of all reported suspected measles cases; 9,123/26,908 (33.90%) were epidemiologically linked, 6,148/26,908 (22.85%) confirmed by laboratory, 2,952/26,908 (10.97%) clinically compatible and 8,685/26,908 (32.28%) were discarded measles cases. The total measles cases (epidemiologically linked, laboratory confirmed and clinically compatible measles cases) were 18,223/26,908 (67.72%). The annual rate of non-measles febrile rash illness cases rate ranged between 1.8 and 4.3 per 100,000 population across the study years. The lowest rates were seen in 2007 and 2016 years, with 1.81 and 1.80 per 100,000 population respectively. Of the 26,908 suspected cases, 15,231 (56.6%) samples were tested by the laboratory for measles virus specific IgM antibody. Of these, 6,148/15,248 (40%) were IgM positive, 8,684/15,231 (32%) were negative for the presence of measles IGM and 399/15,231 (2.6%) had an indeterminate lab result. The highest positive samples for measles IGM were observed in 2015. Of the 9,083 determined to be measles IgM negative or indeterminate cases, 918/8,732 (10.5%) of cases were positive for rubella specific IgM.

**Figure 3:**
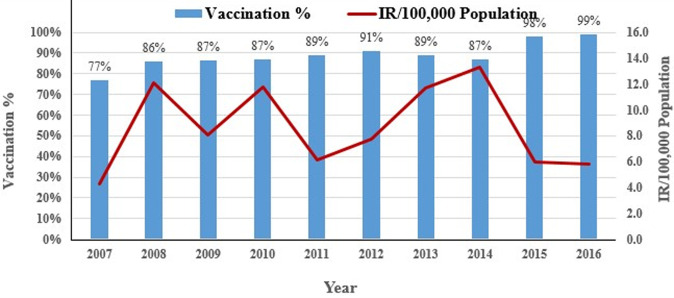
number of measles cases reported and estimated percentage of children who received the first dose of MCV, Oromia 2007-2016

The vaccination status of 12,495/26,908 (46%) cases were found to be of unknown status, while an additional 7,814/26,908 (29%) were not vaccinated. A single dose had been administered in 4,995/26,908 (19%) cases, with 1,088/26,908 (4%) receiving two doses and 516/26,908 (2%) receiving a range of 3-7 doses. West Arsi zone had the highest (19.5%) unknown vaccination status, followed by Arsi zone (8.34%). The highest unvaccinated status were from Guji zone (20%), followed by West Hararge (10.8%). An estimated percentage of children who received the first dose MCV was compared to the number of measles cases reported for each year between 2007 and 2016, however no strong association was observed (Figure 3). Death was significantly associated with the less than five years age group (AOR=1.82, 95%, CI 1.42-2.33), the year range of 2012-2016 (AOR=0.34, 95%, CI: 0.25-0.45), a not vaccinated status (AOR= 1.44, 95%, CI 1.06-1.9), case-based reporting (AOR=0.02, 95%, CI: 0.01-0.04) and inpatient status (AOR=2.19, 95%, CI: 1.58-2.85). Sex and area of residency demonstrated no association with measles death (Table 1).

**Table 1:** association of variables with measles death-Oromia region 2007-2016

Variables	Deaths (N=(%))	Alive (N=(%))	COR (95%CI)	AOR (95%CI)
**Sex**				
Female	148 (51.4)	12507 (46.8)	1.19 (0.9-1.5)	
Male (ref)	140 (48.6)	14113 (53.2)		
**Area**				
Urban	62 (21.5)	5606 (21.06)	0.97 (0.7-1.28)	
Rural (ref)	226 (78.5)	21014 (78.94)		
**Age group**				
>5years	103 (35.76)	15219 (57.1)	0.41 (0.32-0.53)	0.51 (0.40-0.66)
<5 years (ref)	185 (64.24)	11401 (42.8)		
**Data type**				
Case-based	13 (4.5)	17223 (64.7)	0.025 (0.01-0.04)	0.02 (0.01-0.04)
Line list (ref)	275 (95.5)	9397 (35.3)		
**Patient care**				
Outpatient	222 (77.8)	23441 (88.0)	2.19 (1.6-2.8)	1.76 (1.31-2.36)
In patient (ref)	66 (22.9)	3179 (11.9)		
**Reporting year**				
2007-2011	66 (22.9)	12129 (45)	0.35 (0.26-0.4)	0.34 (0.25-0.45)
2012-2016 (ref)	222 (77.1)	14491 (54)		
**Vaccination**				
Vaccinated	53 (18.4)	6546 (25)	1.44 (1.07-1.95)	1.55 (1.14-2.11)
Unvaccinated (ref)	235 (81.6)	20074 (75)		

## Discussion

**Figure 4:**
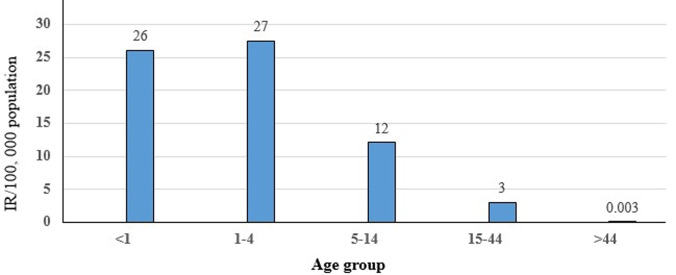
measles incidence per 100,000 population, by age, in Oromia, 2007-2016

The incidence of measles in Oromia region is high and has remained above 5 per 100,000 for the past ten years, with the exception of 2007. This is significantly above the target set by WHO for accelerated measles control of less than 5 per 1,000,000 population [18,19]. The analysis revealed that all age groups were affected, including those older than 15 years, with the majority (85.5%) under 15 years. The median age was 6 years, with an interquartile range of 6 months to 75 years. This suggests that the 1-15 year age group remains an at-risk population for contracting measles in the region. Furthermore, our findings showed that the disease mainly affected children less than five years of age. This finding is supported by a similar measles outbreak investigation conducted in Guji zone of Oromia region, Ethiopia that indicated that under five year of age was the most affected [20]. Similar results were also reported in Uganda, where results indicated 64% of measles cases in children less than five [21]. This same age group was also shown to have the higher mortality when compared to those greater than five years. This result could be due to the fact there is weak immunity during this early age range. Additionally, our research indicated the age specific IR was highest, 27 cases per 100,000 populations, in those aged 1-4 year. Incidence rates then decreased with increasing age to its lowest level (0.003/100,000) in person´s ≥45 years (Figure 4). This finding was similar with a survey done in South Africa [22].

The overall CFR during the ten year study period was 1.07%, which is lower than WHO estimate of 3% to 6% in developing countries [23]. Similarly, this finding is also lower than another studies in Africa [24,25]. The low CFR indicated, however, may be attributed with an under reporting of measles deaths. Further research is needed to elucidate the association. The highest CFR in our study occurred in children aged 1 to 4 years. This is different from the measles surveillance guideline of Ethiopia, which states the highest case-fatality rate occurs in infants 6 to 11 month of age [12]. It was further observed that a high proportion of deaths were reported from 2012-2016 (77.2%), as compared to the 2007-2011 period (22.9%). A number of factors may have contributed to this discrepancy, with likely catalysts being the strengthened performance of surveillance activity and an improvement to the previous under reporting of deaths. The change in the incidence rate and CFR in the region may also reflect the vaccination activities and other measles prevention and control interventions.

The incidence of suspected measles increased from 2007 to 2010, before a remarkable drop was seen in 2011. The rate decreased from 11.7 per 100,000 populations in 2010 to 6.1 per 100,000 population in 2011. However, incidence rates again progressively increased to 13.3 per 100,000 populations in 2014 (Figure 2). The same trend was observed in the mortality rate and incidence of suspected measles during the ten-year study period. This incidence rate may be attributed to the sensitivity of the surveillance system and as a result of an epidemic that occurred within most zones of the region during this period. However, this was not compared specifically to non-outbreak related cases and as a result is only suggestive.

Disparity in the IR was observed among zones in our study. Guji zone was most affected, with an IR of 190/100,000 population, followed by West Arsi zone (IR of 170/100,000 population). These results may be due to difference in early detection and confirmation of the epidemic among zones and higher IRs may be due to the build-up of the susceptible population which may have contributed the spread of the disease faster than expected. Coverage rates may not be as accurate reported as a result of these geographically hard to reach communities. Additionally, portions of both zones contain nomadic pastoral populations making children in these communities difficult to reach [26]. This important demographic is often missed by targeted vaccination campaigns, resulting in the buildup of a susceptible population. It is also possible that the disparity of IR between zones may be the reflection of routine immunization, as the performance regarding routine immunization was shown to vary between zones. Measles transmission increases during the late winter and early spring in temperate climates and after the rainy season in tropical climates [27]. In Ethiopia, peaks of confirmed measles cases occur every year between January and May [28]. In Oromia, a similar seasonal pattern of occurrence of measles has been observed over the years, with highest proportion of measles cases during the 1^st^ quarter of the year (January to March). The number measles cases begin to rise in October, before peaking in January, with the majority of cases occurring during Ethiopia´s dry season. Cases were shown to drop during the rainy months (July, August and September). These results could be due to the school closures in July and August and then reopening in September. This reopening period coincides with the onset of case increases displayed in our study. A similar analysis done in Ethiopia in 2016 revealed a clear peak in transmission of measles overlapping with the start of the school calendar and then a decrease during school closure [16].

To prevent transmission and enhance efforts for elimination of measles the WHO recommends strengthening routine immunization with a two dose-schedule to protect every child and providing an opportunity for measles vaccination through supplementary immunization activities (SIAs) [13]. Administrative coverage data shows differences across Ethiopia´s regions for MCV1 [29]. The administrative coverage of measles vaccination in the Oromia region during the 10 year period ranged from 77% to 99%, with great disparity between zones within the region. In our finding, 29% were not vaccinated and 46.4% were unknown for vaccination status. This seems to indicate that there is a gap in routine immunization coverage. The national epidemiologic surveillance unit is expected to classify all cases following the receipt of laboratory confirmation results. In our analysis, the number of laboratory-confirmed, epidemiologically linked, clinically compatible and discarded cases was seen fluctuating during the study periods, with high incidence of cases following low incidence in study years. The number and percentage of laboratory confirmed measles cases, 441 of 1156 (38%), in 2007 dropped to 302 of 3367 (9.5%) in 2008 and 198 of 2325 (8%) in 2009, before again increasing to 1136 of 2045 (57%) in 2015. The same trend was observed for epidemiologically linked, clinically compatible and discarded cases in the study years.

Sensitivity of a surveillance system refers to the proportion of cases of a disease (or other health-related event) detected by the surveillance system [30]. In 8 of the 10 years of our study, the non-measles febrile rash case rate was greater than 2 per 100,000 population, with the exceptions occurring in 2007 and 2016. This is above minimum WHO African region requirement (target: at least 2 per 100,000 population per year) [31] and suggests that strengthening of measles surveillance is needed. Our finding showed 40% of tested samples in this surveillance system had positive results for measles IgM. This result was higher than similar studies done in Ethiopia´s Amhara and Southern Nations Nationalities and Peoples (SNNP) regions [28,32] within a similar timeframe. The number and percentage of positive and negative measles IGM results were observed fluctuating throughout the years. Higher positive measles IGM findings were seen in 2015 (56%) followed by 2014 (52%). Higher negative measles IGM seen in 2009.

Conducting case-based measles surveillance is a target of the WHO regional measles elimination goal to be achieved by 2020 [33]. Ethiopia implemented a system of case-based surveillance for measles staring in 2005 [29]. The majority (64%) of suspected measles case were reported through this case-based reporting and shown to be higher during 2012-2016, as compared to the reports in 2007-2011. This may be the reflection of strengthened surveillance activities performed to achieve the set objectives put in place to meet the measles elimination goal. Line list data surveillance was also conducted during this study. Regression analysis indicated a significant relationship is shown between line list reporting and measles deaths, however, this output must be discounted as the technique was not utilized in 2011 and 2015. As a result, further investigation is needed into the impact of surveillance data type and its relationship with measles deaths. This study did incur some additional limitations. Due to the nature of study design, in that we were relying on secondary surveillance data, the method of obtaining vaccination status of cases was unknown, thus not described. It is also possible that the case fatality of measles rate may be underestimated due to potential under reporting of measles deaths. Furthermore, vaccination coverage data relied solely on administrative reporting.

## Conclusion

Despite vaccination efforts, measles is still a public health problem in the Oromia region of Ethiopia. The burden of the disease remains remarkably high and it persists in most of Oromia zones each year. Demographically, we identified those under the age of 5 as the most affected population, as well as the age group most significantly associated with measles deaths. Other factors shown to be associated with a higher risk of mortality were treatment at the inpatient level and an unvaccinated status. Mortality was also shown to be more likely during the years of the study period ranging from 2012-2016. This time period contained the highest IR year, 2014, (13.35/100,000) which may have influenced this outcome. The first quarter of the year consistently represented the highest transmission period, with January serving as the most prominent month. These outcomes suggest that the immunization program within Oromia needs to continue to be strengthened to reach all children. The use of the MR vaccine should also be considered as a result of the 10% measles negative cases that are positive for rubella. Further investigations directed at understanding the incidences rate and case detection rate disparities across zones are also recommended.

### What is known about this topic

- In Ethiopia, measles has been one of the major causes of death and sickness of children;
- The Oromia region of Ethiopia is particularly impacted by measles with 3,507 suspected cases of measles were reported from 2005-2009;
- Those under the age of 5 are at more likely to suffer from measles complications.

### What this study adds

- Despite vaccination campaigns, the burden of measles remained high over the 10-year study period in the Oromia region of Ethiopia;
- Of the cases determined as measles IgM negative or indeterminate cases, 10.5% were positive for rubella specific IgM suggesting the use of the MR vaccine be considered;
- All areas of the Oromia region were not uniform in terms of infection rate. The highest IR was observed in the boarding zones of Guji and West Arsi (central/southern Oromia).
